# Response to ‘Comment on “Evaluation of Micronucleus in Exfoliated Human Buccal Epithelium Cells among E-Waste Exposed Residents in Payatas, Philippines”’

**DOI:** 10.5696/2156-9614-11.29.210310

**Published:** 2021-02-25

**Authors:** Julie S. Berame, Zeba F. Alam, Frosyl Miguel, Liz Noguera, Aris Lapada

**Affiliations:** 1 Education/Biology Department, Caraga State University, Ampayon, Butuan City, Philippines; 2 Biology Department, De La Salle University, Manila, Philippines; 3 Science and Technology Department, Ramon Magsaysay High School, Manila, Philippines; 4 Science Department, Manuel Roxas High School, Manila, Philippines; 5 Biology Department, Eastern Samar State University, Borongan City, Philippines

We express our appreciation of the response to our micronucleus paper^1^ and are glad that our research findings are relevant to other findings on e-waste issues from the plethora of literature that we have read. We understand that there are confounding factors to consider when it comes to micronucleus assays. Based on our reading of other related studies, the use of buccal micronucleus assay for biomonitoring of e-waste workers is definitely possible because the micronucleus (MN) assay in exfoliated buccal cells is a useful and minimally invasive method for monitoring genetic damage (as also experienced among e-waste workers) in humans. The MN assay in buccal cells has been used since the 1980's to demonstrate the cytogenetic effects of environmental and occupational exposures, lifestyle factors, dietary deficiencies, and different diseases, but important knowledge gaps remain about the characteristics of micronuclei and other nuclear abnormalities, with the basic biology explaining the appearance of various cell types in buccal mucosa samples and effects of diverse staining procedures and scoring criteria in laboratories around the world. With these uncertainties, the human micronucleus project (HUMN)^2^ has initiated a new international validation project for the buccal cell MN assay. They advised that future research should explore sources of variability in the assay (e.g., between laboratories and scorers, as well as inter- and intra-individual differences in subjects), and resolve key technical issues, such as the method of buccal cell staining, optimal criteria for classification of normal and degenerated cells and for scoring micronuclei and other abnormalities. The harmonization and standardization of the buccal MN assay will allow more reliable comparison of data among human populations and laboratories, evaluation of the assay's performance, and consolidation of its world-wide use for biomonitoring of DNA damage.^3^

In this regard, the buccal cell MN assay was first proposed in 1983 and continues to gain popularity as a biomarker of genetic damage in numerous applications. More than 40 laboratories from many countries either have used or are currently using this assay, and the number of articles published annually is steadily increasing. Different issues related to the buccal cell MN assay were reviewed in several publications over the last decade.^4^

Since the publication of the previous e-waste monitor in 2017, the number of studies on the adverse health effects from e-waste have increased. These studies have continued to highlight the dangers to human health from exposure to well-studied toxins, such as lead. Recently, research has found that unregulated e-waste recycling is associated with increasing numbers of adverse health effects. These include adverse birth outcomes, altered neurodevelopment, adverse learning outcomes,^5^ DNA damage,^6^ adverse cardiovascular effects,^7^ adverse respiratory effects,^8^ and adverse effects on the immune system.

Additionally, the Micronucleus Assay Expert Group reported some confounding factors when performing the MN assay using buccal cells, such as age, smoking habits, gender, along with criteria for categorizing participants as smokers. In our study, we adopted the checklist questionnaire from the Life Interview of Smoking Trajectories and Quitting Methods Questionnaire on smoking histories developed by the Methods and Measurement core of the Brown-Harvard Transdisciplinary Tobacco Use Research Center.^9^ This instrument obtains information on participants' experiences with smoking by gathering of data on regular smoking, levels of consumption, and more. Regular smoking was defined as a positive response in our micronucleus study conducted in Payatas, Philippines.^1^ We created a summary measure of cigarette consumption using data on participants' smoking intensity. Participants also provided information on the age of onset of regular smoking and initial age of smoking. This information is important, but the latest data suggest that there is a difference between the percentage of cells with micronuclei in smokers with a smoking history of less or more than 10 years, but this is not significant.^10^ Although it is important to investigate the synergistic impact of these lifestyle variables, particularly with respect to smoking and e-waste toxicity, these variables can only have impacts if exposures occur over a long period of time, both in terms of smoking as well as e-waste recycling.

It is correct that cigarette smoking can induce metanuclear changes in oral mucosa cells,^8^ but based on our data collected from non-smoker participants, we counted micronuclei as equivalent to the micronuclei counts from smoking participants. It is also claimed that buccal epithelial cells are the direct route of exposure to different types of genotoxic agents. The MN assay is most frequently used for tobacco-associated buccal cell abnormalities. Several studies have indicated that smoking habits elevated micronuclei frequencies, but others have not found any effects with respect to the occurrence of micronuclei in buccal mucosa cells.^11^

With regard to Giemsa staining, the literature survey shows that Giemsa stain has been used in a number of studies,^12^ in spite of it being a less specific stain compared to DNA-specific stains such as Feulgen, acridine orange, 4',6-diamidino-2-phenylindole (DAPI) and propidium iodide, although some of these DNA specific stains have been identified as genotoxic and capable of causing DNA damage. Furthermore, the frequency of micronuclei (FMic) in Giemsa-stained slides have been found to be correlated with genetic polymorphisms, corroborating that the applied stain method in our study is valid.^13^ We are aware that the human micronucleus (HUMN) collaborative programme^14^ tried to unify the protocol for the MN essay. It was observed that there were no major discrepancies with respect to the inter-laboratory slide scoring exercise and the analyses of data for methodological, demographic, genetic, lifestyle and exposure variables.^14^ None of these recent studies included any objection to the use of Giemsa stain for micronuclei studies and research papers on micronuclei with Giemsa stain have been published in many reputable and Scopus-indexed journals.

**Abbreviations**
*MN*: Micronucleus
*FMic*: Frequency of micronucleus

In the e-waste toxicity studies, the researchers hope to identify more e-waste recyclers located at other e-waste dumpsites that have been engaged in e-waste recycling for longer periods of time. Investigation of the genotoxicity of e-waste in terms of the inverse relationship between cytotoxicity and MN frequency will become clear only once we gather further data on long-term exposures and compare it with e-waste recyclers that have fewer years of exposure.^5^ Furthermore, the age of the recyclers and other co-morbidities can have an impact on the results as well. Therefore, at the moment, the researchers feel that this is outside of the scope and data of the present study. In the future, we do intend to take his suggestion into consideration and carry out further studies to investigate the cytotoxicity and incidence of MN in e-waste recyclers.

We do not agree that Fig. 2A and 2B show any discrepancies due to the use of Giemsa stain in terms of keratinocytes interfering in the scoring of micronuclei. A strict scoring criterion was used and followed which was a very time-consuming process and in case of any discrepancy, the cells and sometimes entire slides were not used for scoring purposes to avoid any inconsistencies.

This is not the first micronuclei study that has been conducted by our laboratory. We have sufficient experience in using micronuclei testing in other cells and model systems as well and follow the guidelines stipulated for the identification of micronuclei. As stated in our response to the previous query, no major inter-laboratory discrepancies have been reported in terms of scoring or identification of micronuclei, and with our experience in this field, we want to confirm that due diligence and utmost care has been taken while scoring the slides.

Finally, we are thankful for his comments and suggestions, but the epidemiological studies conducted with respect to e-waste recyclers are very difficult to carry out as there are many lifestyle-related variables such as malnutrition, smoking, alcoholism, and more that are prevalent in this section of society. Therefore, the possibility of synergistic effects of these factors along with the e-waste-induced toxicity needs further investigation. Another serious issue which the researchers face is the reluctance of e-waste recyclers to identify and acknowledge their involvement in the recycling profession due to fear of losing their jobs in case the attention of the NGOs, environmentalists, and other welfare organizations are directed towards the closure of informal recycling activities. Hence, collection of information, data and samples have their own challenges. We hope to continue this research as we know that e-waste generation in the near future is going see a tremendous increase, especially due to our increased dependency during this ongoing pandemic. We want to assure our fellow scientific community that this research has been conducted correctly, guided by a plethora of studies and literature and with the aim of finding ways to protect e-waste recyclers from the genotoxic effects of e-waste.
